# Serum response factor regulates smooth muscle contractility via myotonic dystrophy protein kinases and L-type calcium channels

**DOI:** 10.1371/journal.pone.0171262

**Published:** 2017-02-02

**Authors:** Moon Young Lee, Chanjae Park, Se Eun Ha, Paul J. Park, Robyn M. Berent, Brian G. Jorgensen, Robert D. Corrigan, Nathan Grainger, Peter J. Blair, Orazio J. Slivano, Joseph M. Miano, Sean M. Ward, Terence K. Smith, Kenton M. Sanders, Seungil Ro

**Affiliations:** 1 Department of Physiology and Cell Biology, University of Nevada School of Medicine, Reno, Nevada, United States of America; 2 Department of Physiology, Wonkwang Digestive Disease Research Institute and Institute of Wonkwang Medical Science, School of Medicine, Wonkwang University, Iksan, Chonbuk, Korea; 3 Aab Cardiovascular Research Institute, University of Rochester School of Medicine and Dentistry, Rochester, New York, United States of America; Cinvestav-IPN, MEXICO

## Abstract

Serum response factor (SRF) transcriptionally regulates expression of contractile genes in smooth muscle cells (SMC). Lack or decrease of SRF is directly linked to a phenotypic change of SMC, leading to hypomotility of smooth muscle in the gastrointestinal (GI) tract. However, the molecular mechanism behind SRF-induced hypomotility in GI smooth muscle is largely unknown. We describe here how SRF plays a functional role in the regulation of the SMC contractility via myotonic dystrophy protein kinase (DMPK) and L-type calcium channel CACNA1C. GI SMC expressed *Dmpk* and *Cacna1c* genes into multiple alternative transcriptional isoforms. Deficiency of SRF in SMC of *Srf* knockout (KO) mice led to reduction of SRF-dependent DMPK, which down-regulated the expression of CACNA1C. Reduction of CACNA1C in KO SMC not only decreased intracellular Ca^2+^ spikes but also disrupted their coupling between cells resulting in decreased contractility. The role of SRF in the regulation of SMC phenotype and function provides new insight into how SMC lose their contractility leading to hypomotility in pathophysiological conditions within the GI tract.

## Introduction

Serum response factor (SRF) is a ubiquitous expressed transcription factor that drives smooth muscle cell (SMC)-specific gene expression and is necessary for contractile and cytoskeletal functions. SRF transcriptionally activates the expression of SMC-specific genes by binding to CArG [CC (A/T)_6_ GG] boxes in promoters and introns of most SMC-restricted genes [1]. Computational analysis of genome-wide CArG boxes (CArGome) in mice and humans has identified many SRF-dependent genes that encode for cytoskeletal/contractile or adhesion proteins suggesting that SRF is an ancient master regulator of the actin cytoskeleton [2].

SRF is essential for the growth and differentiation of SMC in the gastrointestinal (GI) tract. Depletion of SRF in SMC, in *Srf* deficient mice, results in a dramatic decrease of contractile function, the degeneration of smooth muscle, and severe dilation of the GI tract [3–5]. However, it remains unclear how SRF regulates physiological contractile function of SMC in the GI tract.

We have previously built the Smooth Muscle Genome Browser linked to the UCSC Genome Browser (UCSC Smooth Muscle Genome Browser) that shows genome scale transcriptional expression data and SRF binding sites (CArG boxes) obtained from mouse jejunal and colonic SMC: http://med.unr.edu/physio/transcriptome. Both jejunal and colonic SMC expressed genes into multiple transcriptional variants, of which most appeared to be specific to SMC [6]. This browser offers a new perspective into the alternative expression of genes in the context of SRF binding sites in SMC and provides a valuable reference for future functional studies [6].

In GI smooth muscle, the activation of Ca^2+^-activated Cl^−^channels in the interstitial cells of Cajal (ICC) produces electrical slow waves, which are conducted to SMC to produce cycles of depolarization [7–9]. Depolarization of SMC activates Ca^2+^ channels, which allows Ca^2+^ entry to increase intracellular calcium concentrations [Ca^2+^]_i_ [10, 11]. This excitation-contraction coupling of smooth muscle is mainly regulated by voltage-activated L-type Ca^2+^ channels [12].

SMC express the α1C subunit of L-type Ca^2+^ channels (CACNA1C) [13], and a recent study showed that myotonic dystrophy protein kinase (DMPK) regulates transcriptional expression and alternative splicing of the α1S subunit of L-type Ca^2+^ channels (CACNA1S) in skeletal muscle [14]. DMPK is expressed in all major muscles including smooth, skeletal, and cardiac muscles [15] and is linked to myotonic dystrophies [16]. Furthermore, DMPK regulates activities of the multiple proteins within Ca^2+^ signaling pathways in muscle cells. These activities include sarcoplasmic uptake of Ca^2+^, smooth muscle Ca^2+^ desensitization, and cytoskeletal rearrangements [17]. However, it is still unknown whether a transcriptional factor is involved in driving the muscle-specific expression of DMPK or whether DMPK regulates the excitation-contraction coupling.

We report here a model for the functional role of SRF that involves regulation of SMC contractility via SRF-induced DMPK and its downstream target, the L-type calcium channel CACNA1C. Our proposed model offers new insight into how loss of SRF expression can lead to functional changes in SMC in the pathogenesis of GI motility disorders.

## Materials and methods

### Animal care

All animal use procedures were reviewed and approved by the Institutional Animal Care and Use Committee at the University of Nevada, Reno (UNR). UNR is fully accredited by AAALAC International.

The colony of laboratory mice included in this experiment were housed in a Centralized Animal Facility at the University of Nevada-Reno Animal Resources. All were animals housed in individually ventilated, HEPA-filtered microisolator cages (Tecniplast) under positive pressure relative to the room. Cages were sanitized in accordance with the Guide for the Care and Use of Laboratory Animals (National Research Council, 2011). Ultra-purified water was provided ad libitum. The diet was irradiated mouse chow (Harlan Teklad 2919) and cage enrichment was provided to all animals. Sentinel mice are tested quarterly for potential pathogens [IDEXX BioResearch (Columbia, MO) is used as the reference diagnostic laboratory].

The animals were checked twice daily by research personnel and the animal care staff. Pain assessment was done using the Grimace Scale published by the National Centre for the Replacement, Refinement and Reduction of Animals in Research (NC3Rs). End points were determined when the animals exhibiting moderate pain which is a score of “1” on NC3Rs Grimace Scale. Analgesics were not administered during these experiments. Animals were euthanized by CO_2_ inhalation overdose in accordance with the 2013 guidelines by the American Veterinary Medical Association.

### Generation of inducible *Srf* knockout mice

The SMC-specific inducible *Srf* knockout (KO) mouse line *Tg(Myh11-Cre-ERT2);Srf*^*lox/lox*^ was generated by cross-breeding a *Srf*^lox/lox^ female homozygote mouse (The Jackson Laboratory, Bar Harbor, ME) with a *Tg(Myh11-Cre-ERT2)* male mouse [18] according to procedures approved by the Institutional Animal Care and Use Committee at the University of Nevada, Reno. Mouse pups at the ages of 15–20 days were genotyped using primer sets by PCR (for primer sequences, see S1 Table). Briefly, a small tail tip was amputated, genomic DNA was isolated from the tissue, and PCR was performed with genotyping primer sets. Primers are: SRF-gt1 and SRF-gt1r for lox site insertion, Myh-I1, Myh-E2r, and Cre-r for Myh11 gene and Cre-ERT2 cassette. Deletion of the *Srf* promoter and exon 1 was confirmed in genomic DNA from *Srf* KO smooth muscle tissue by PCR. Primers are: SRF-gt1 and SRF-gt2r for the lox deletion.

### Tamoxifen injection

Tamoxifen (Sigma, St Louis, MO) was diluted in 10%/90% vol/vol ethanol/oil to a concentration of 1 mg/100 μl. Groups of homozygous *Srf* KO [3–4 weeks old, *Tg(Myh11-Cre-ERT2;Srf*^*lox/lox*^)] were treated with daily intraperitoneal (IP) injections of tamoxifen (1 mg/day), or of solvent only (ethanol/sunflower oil for a control group), for 5 consecutive days.

### Tissue preparation

The transgenic KO and wild type (WT) mice at post tamoxifen injection days 5 (KO5D), 10 (KO10D), 15 (KO15D) and 20–21 (KO20/21D), were anesthetized by isoflurane inhalation prior to sacrifice at multiple time points of developmental ages. Stomach, small intestine, and colon were dissected from the mice. Whole jejunum was used for H&E staining, immunohistochemistry and confocal microscopy. Smooth muscle, which was stripped free of mucosa and submucosal plexus, was used for reverse-transcription polymerase chain reaction (RT-PCR), quantitative PCR (qPCR), proteomics, Western blot, mechanical study, and Chromatin Immunoprecipitation (ChIP) qPCR analysis. Strips of longitudinal muscle that were partially dissected from small intestines were used for calcium imaging.

### Isolation of total and small RNAs

Total RNAs were isolated from jejunum and colon from *Srf* KO and WT mice as previously described [19]. The extracted total RNAs were used for gene expression analyses using RT-PCR and/or qPCR.

### RT-PCR and qPCR detection

Preparation of the cDNA libraries from the total RNAs, as well as RT-PCR and qPCR analysis on cDNAs, were performed as previously described [20]. All primers used for RT-PCR and qPCR are shown in S1 Table. All RT-PCR and qPCR products were analyzed on 1.5% agarose gels.

### Proteomics analysis

Proteins prepared from jejunum of WT and *Srf* KO mice at KO15D (n = 6) were analyzed by two-dimensional LC-MS/MS on a Thermo Finnigan LTQ-Orbitrap (Thermo Scientific, Fremont, CA) and identified using Ingenuity Pathway Analysis (IPA, QIAGEN, Redwood City, CA) as previously described [5].

### Bioinformatics analysis

Genes of mRNAs regulated by SRF were analyzed using IPA software. IPA analyzed relationships among down-regulated proteins and SRF. Genomic location of exons, CArG boxes, and SRF binding sites in the genes of transcriptional variants were analyzed using the newly constructed UCSC Smooth Muscle Genome Browser [6].

### Western blots

Protein extract preparation from tissue samples and western blotting were performed as described previously [21]. Primary antibodies against the following antigens were used: CACNA1C (guinea pig, 1:1000, Alomone Labs, Jerusalem, Israel), SRF (rabbit, 1:500, Santa Cruz Biotechnology, Dallas, TX), or GAPDH (rabbit, 1:1500, Cell Signaling Technology, Danvers, MA). All antibodies were incubated in 5% Blotto, non-fat dry milk (Santa Cruz Biotechnology, Dallas, TX). HRP-conjugated goat anti-guinea pig (AP108P), goat-anti-rabbit (AP307P), or goat anti-mouse (AP308P) secondary antibodies (1:50,000; EMD Millipore, Billerica, MA) were used with an enhanced chemiluminescence method (Amersham ECL Advantage, GE Healthcare Life Sciences, Pittsburgh, PA). The protein bands were captured using a CCD-camera system (EC3 410 Imaging System; UVP, Upland, CA) and analyzed with VisionWorksLS software (Version 6.8; UVP, Upland, CA). The expression level of CACNA1C and SRF was normalized to the ratio of CACNA1C and SRF area density divided by GAPDH area density. For quantitative measurements, the values of relative expression for CACNA1C and SRF in KO5D, KO10D, and KO15D small intestine were compared to the expression in WT10D small intestine (set to 1.0).

### Confocal microscopy and histological analysis

Tissues were analyzed by cryostat section staining using confocal microscopy as previously described [5]. Jejunum and colon tissues were double stained with anti-SRF (1:500, Santa Cruz Biotechnology, Dallas, TX) and anti-αSMA (1:1200, Sigma, St. Louis, MO) antibodies. Stained tissues were analyzed using confocal microscopy. For histological analysis, tissues were dehydrated, embedded in paraffin, cut into 4 μm-thick coronal sections, rehydrated and stained with H&E. Images were collected using an Olympus FV1000 confocal laser scanning microscope with Fluoview FV10-ASW 3.1 Viewer software (Olympus, Tokyo, Japan) or the iScan Coreo scanner (Ventana Medical Systems, Tucson, AZ).

### Analysis of mechanical response

Mechanical contractile activity was measured using standard organ bath techniques at KO15D-18D. The mucosa was removed from antrum and colon tissues. Strips (10 mm x 3 mm) were then cut from the underlying smooth muscle. These strips were cut parallel to the circular muscle layer and a fine silk suture thread was tied to each end. One end was then tied to a fixed point in an organ bath (7 ml) that was continuously perfused with oxygenated KRB solution maintained at 37°C. The other end was tied to a FORT 10 force transducer (World Precision Instruments, Sarasota, FL). Isometric force was recorded in the organ baths before and after addition of 1 μM Bay K8644, 36 mM K^+^ and 72 mM K^+^ as described previously [22]. To analyze the gathered data, the area under the trace (AUT) was calculated as a measurement of contractile activity. AUT was determined under control conditions and after the addition of Bay K8644, 36 mM K^+^ and 72 mM K^+^. For each condition, the fold increase, compared to control, was calculated. Subsequently, we compared the fold changes in WT and KO tissues using the unpaired Student’s t test. Determining the fold change for WT and KO tissues allowed us to take into account the fact that KO animals have a significantly reduced smooth muscle layer.

In other experiments, whole tissue segments were secured to the Sylgard (Dow Corning, Midland, MI) lined floor of an organ bath (30 ml) by pinning the mesentery. A silk suture was used to connect a force transducer (model TST125C; Biopac Systems Inc., Goleta, CA) along the colon. Resting tension was initially set at 5 mN and the bath was perfused with oxygenated Krebs solution (37°C) throughout the experiment. Nω-Nitro-L-arginine (L-NNA) (100 μM) and atropine (1 μM) were added to the Krebs solution and perfused into the organ bath.

In both types of experiment, contractions were monitored using an MP100 interface and recorded on a PC running Acqknowledge software 3.9.2 (Biopac Systems Inc., Goleta, CA). The composition of the Krebs solution was (mM) 120.35 NaCl, 5.9 KCl, 15.5 NaHCO_3,_ 1.2 NaH_2_PO_4_, 1.2 MgSO_4_, 2.5 CaCl_2_, and 11.5 glucose at 37°C.

### Calcium imaging

Duodenal and jejunal segments (20–30 mm long) at KO15D-18D from age-and sex-matched WT controls were cut longitudinally along the mesenteric border, and pinned flat with the serosa-side up. Longitudinal muscle strips were peeled away by sharp dissection to expose the underlying myenteric plexus. Fluo-4 AM (Thermo Scientific, Fremont, CA) was incubated with tissue in order to measure the elevations of [Ca^2+^]i of ICC and SMC. Recordings were analyzed by in-house analysis software which generated fluorescence intensity traces by stabilizing image sequences.

After an equilibration period (30 minutes), isolated flat-sheet preparations were loaded with 25 μg of Fluo-4 (FluoroPure™-AM, Molecular Probes, Eugene, OR) in a solution of 0.02% DMSO and 0.01% non-toxic detergent Cremophor EL for 20 minutes at 25°C. The dye was preferentially loaded into the outer LM layer, as the mucosa provided a barrier to the solution to keep it from diffusing through or underneath these preparations. After incubation, the preparation was perfused with warm KRB solution for 20 minutes to allow for de-esterification of the dye. For anesthetized mice, the dye solution was applied directly to the exteriorized loop for 15 minutes at 37°C. Following loading, the exteriorized loop was rinsed with warm KRB solution for 5 minutes, followed by 15 minutes equilibration to allow for de-esterification of the dye [23, 24].

Functional imaging was performed on a Nikon Eclipse E600FN upright fluorescence microscope using Nikon Fluor water immersion lenses (20–60×, Nikon, NY). The fluo-4 indicator was excited at 488nm using a Lambda LS xenon illuminator (Sutter Instrument Co., Novato, CA) and a modified GFP dichroic cube (ex:488, em: 543; Chroma Technology Corp., Bellows Falls, VT), and image sequences were captured using either a Photometrics Cascade 512-B camera (Roper Scientific Inc., Trenton, NJ) or an Andor iXon +897 (Andor Technology, Belfast, UK) EMCCD camera. Image sequences (1000–2000 frames, 15.67–32.4 Hz) were captured on a Windows-based PC using Metamoph 6.16 (Molecular Devices, Sunnyvale, CA) for the 512-B, or Andor Solis 4.14 (Andor Technology) for the iXon +897 [23, 24].

Raw Metamorph (16-bit,.stk) and Solis (16-bit,.tif) files were analyzed on a PowerMac G5 desktop computer (Apple Inc., Cupertino, CA) using in-house analysis software (Volumetry G6a, G. W. Hennig). Movements of the preparation were tracked and algorithms to allow for stable measurements from individual regions of interest (ROIs) were used. Ca^2+^ induced fluorescence is reported as an average intensity inside rectangular ROIs in 8-bit intensity units [23, 24].

### Chromatin Immunoprecipitation (ChIP) qPCR analysis

ChIP assays were performed using the ChIP-IT High-Sensitivity Kit (Active Motif, CA). Approximately 70 mg of freshly dissected jejunal smooth muscle from the *Srf* KO male mice and WT male sibling at KO15D and WT15D was minced finely and cross-linked with 1% formaldehyde (Sigma-Aldrich, MO) for 10 minutes at room temperature, quenched with 125 mM glycine for 5 minutes, and then prepared for chromatin sonication according to the manufacturer’s specifications. Chromatin was sheared by sonication using UCD300 Bioruptor (Diagenode, NJ). Sonicated chromatin was incubated overnight with antibodies (4 μg per 30 μg chromatin) against RNA pol II (Active Motif, 39097), SRF [G-20 (2 μg, sc-335) and H-300 (2 μg, sc-13029), Santa Cruz Biotechnology, TX], and respective control IgGs. Before immunoprecipitation, 5% of the extract volume was removed and served as an input. The antibody-bound protein/DNA complexes were immunoprecipitated through the use of protein G agarose beads and washed via gravity filtration. Following immunoprecipitation, the DNA cross-links were reversed and the DNA was purified according to the manufacturer’s protocol. Purified immunoprecipitated and input DNAs were used in qPCR reactions on a 7900HT Fast Real-Time PCR System (Applied Biosystems, CA). The primers for ChIP qPCR analysis are shown in S1 Table. Data were expressed as fold enrichment of the ChIP samples relative to the IgG samples.

### Statistical analysis

All the data obtained in the present study were compared using the paired (qPCR and thickness measurement) or unpaired (analysis of mechanical response) Student’s t test to determine whether the differences were statistically significant. The means of normalized values from at least three independent experiments were determined and subjected to Student’s t test.

## Results

### Identification of SRF-induced proteins, including DMPK, in *Srf* KO SMC

**Fig 1:**
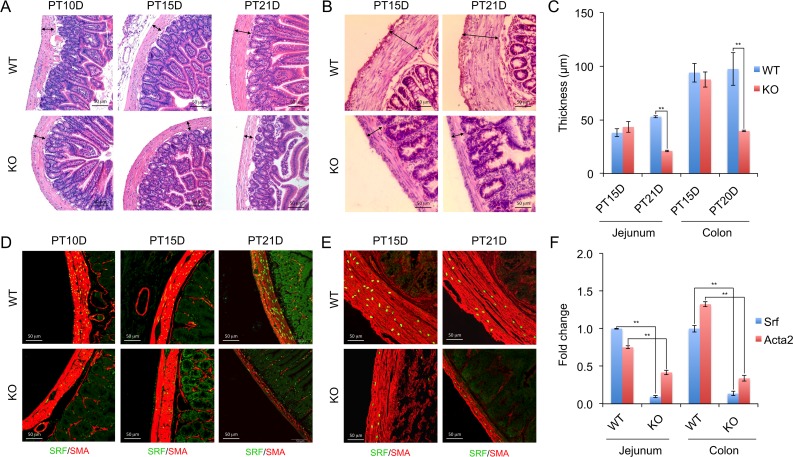
Smooth muscle degeneration in inducible SMC-specific *Srf* KO intestine. (A and B) Cross sectional images of WT and KO jejunum (A) and colon (B) with H&E staining 10, 15 and 21 days post-tamoxifen injection (PT10D, PT15D, and PT21D). Note that KO smooth muscle layers (arrows) are significantly thinner at PT15D and/or PT21D compared to WT control. (C) Summary of thickness of smooth muscle layers in KO and WT. (D and E) Cryosection images of jejunum (D) and colon (E) stained with anti-SRF and anti-SMA antibodies showing gradual depletion of SRF (green, nucleus) and reduction of SMA (ACTA2, red, cytoplasm) in KO SMC compared to WT control at PT10D, PT15D, and PT21D. (F) qPCR analysis to validate reduction of *Srf* and *Acta2* in KO jejunum and colon at PT15D. *Ubb* was used as an endogenous control. Each data point (C and F) represents the mean ± SD of experiments (n = 3). * *p* ≤ *0*.*05* and ** p ≤ *0*.*01*, WT versus KO.

To study the functional roles of SRF in GI SMC, we generated a tamoxifen-inducible SMC-specific *Srf* KO mouse line [5]. Following tamoxifen induced KO, the mice developed progressive and severe degeneration of smooth muscle in the GI tract at around 21 days post-tamoxifen injection (PT21D) (Fig 1A and 1B). Degeneration of smooth muscle in the colon began earlier at PT15D than in jejunum by PT21D. The thickness of jejunal and colonic smooth muscle was differentially changed at PT15D: it was slightly increased in jejunal smooth muscle while it was slightly decreased in colonic smooth muscle. However, both smooth muscles were significantly reduced at PT20-21D in *Srf* KO mice (Fig 1C). Depletion of SRF protein in *Srf* KO SMC were confirmed as SRF expressing SMC were significantly reduced as early as PT10D (Fig 1D and 1E). The longitudinal and circular muscle layers were relatively intact at KO PT10D and PT15D, but they were severely lost at KO PT21D, compared to wild type (WT) muscle layers (Fig 1D and 1E). This phenotypic degeneration of SMC in *Srf* KO was consistent with our previous SRF-induced microRNA study [5]. We validated reduced expression of *Srf* and smooth muscle actin gene (*Acta2*, known as SMA) by qPCR. *Srf* transcripts in KO jejunum were almost completely depleted at PT15D (Fig 1F), consistent with the immunohistochemistry data (Fig 1D and 1E). *Acta2* is a SRF target gene, whose expression is induced by SRF via CArG boxes [25]. *Acta2* transcripts were also significantly reduced in KO jejunum at PT15D, confirming SRF deficiency-mediated reduction of the gene expression in the animal (Fig 1F).

**Fig 2:**
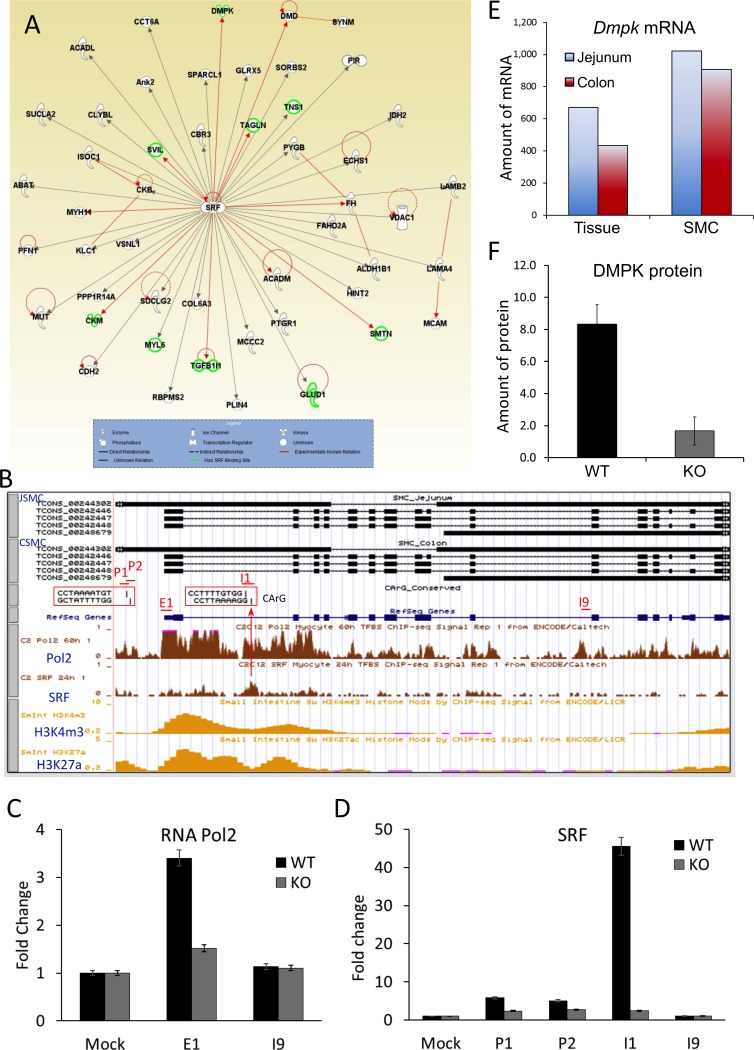
Identification of SRF-induced DMPK that regulates expression of *Celf1* and *Mbnl1*. (A) Interactions between down-regulated proteins and SRF were analyzed using Ingenuity Pathway Analysis (IPA) software. Note that experimentally known relationships are indicated by a red line and genes containing SRF binding site(s) are indicated by highlighted proteins in green. (B) A genomic map of *Dmpk* variants expressed in SMC of jejunum and colon shown on the UCSC Smooth Muscle Genome Browser. SRF binding sites and CArG boxes are indicated in upstream promoter region and intron 1. ChIP-seq data of RNA polymerase 2 and SRF from C2C12 myocytes, and of H3K4m3 and H3K27a from mouse small intestine, are shown under the genomic map. Primer sets spanning regions of the promoter (P1 and P2), promoter and exon 1 (E1), and introns (I1 and I9) of *Dmpk* are indicated. (C) ChIP qPCR data analysis of RNA polymerase 2. DNA fragments isolated from *Srf* WT and KO jejunum smooth muscle (n = 3, respectively) by RNA polymerase 2 antibody were used for ChIP qPCR performed with primer sets spanning regions of E1 and I9 of *Dmpk* shown in *B*. Mock is negative control DNA captured with IgG. (D) ChIP qPCR data analysis of SRF. SRF ChIP and qPCR were performed as the same as above except using an anti-SRF antibody and primer sets spanning regions of CArG boxes (P1, P2, and I1), and a downstream intron region (I9) in *Dmpk* as a negative control. (E) Enrichment of *Dmpk* mRNAs in SMC of jejunum and colon. Amount of mRNAs expression (normalized FPKM) was obtained from RNA-seq data [6]. (F) Reduced expression of DMPK protein in *Srf* KO smooth muscle. Amount of protein expression was obtained from LC-MS/MS proteomics data.

To identify SRF-regulated proteins in SMC, we performed a proteomics study on *Srf* KO and WT jejunum smooth muscle using multidimensional protein identification technology. This proteomic analysis identified 82 down-regulated proteins in KO SMC (S2 Table). A pathway analysis of the proteins using IPA software further identified 50 proteins that interact with SRF (Fig 2A), which consisted of 19 enzymes, 2 transcriptional regulators, 2 kinases, 2 phosphatase, 1 ion channel, and 24 unknown proteins. Nine of these proteins (DMPK, SVIL, MYH11, CKM, TGFB1I1, SMTN, FH, TAGLN, and DMD) were already known to be modulated by SRF (red lines, Fig 2A). We also identified 9 genes (*Dmpk*, *Svil*, *Tns1*, *Ckm*, *Myl6*, *Tgfb1i1*, *Glud1*, *Smtn*, *Tagln*) that contained SRF binding sites at either promoter or intronic regions (S3 Table) using the SRF binding sites published in ENCODE/Caltech, which were obtained from myocyte C2C12 cells through SRF ChIP-seq [26]. A search for CArG boxes in the SRF binding sites of the 9 aforementioned genes revealed that all genes except *Glud1* contained CArG boxes, which were conserved between mice and humans (S3 Table).

We focused our attention on dystrophia myotonica protein kinase (DMPK) for further analysis since it regulates the expression of L-type calcium channels in skeletal muscle [14]. Using the UCSC Smooth Muscle Genome Browser [6], we discovered that both jejunal and colonic SMC expressed three splice variants of *Dmpk* as well as an antisense transcript (Fig 2B). There are four CArG boxes in an upstream promoter region (CCTAAAATGT and GCTATTTTGG) and intron 1 (CCTTTTGTGG and CCTTAAAAGG) of the gene, which were conserved in mice and humans. However, SRF ChIP-seq data from C2C12 cells [27] showed SRF binds to the second CArG boxes at intron 1. RNA polymerase 2 ChIP-seq data from C2C12 cells [27] showed increased binding activities around the promoter, exon 1, and intron 1 of *Dmpk*, which are matched with the gene activation signals of epigenetic histone modification markers H3K4m3 and H3K27a in the small intestine [27]. Amenable to the ChIP-seq data, RNA polymerase 2 ChIP qPCR data obtained from *Srf* WT jejunum showed increased binding activity around the proximal promoter and exon 1 of the gene, compared to the downstream intron 9 and negative mock control (Fig 2C). Interestingly, the increased activity was abolished in the *Srf* KO jejunum, suggesting that SRF is required for the binding of RNA polymerase and transcriptional elongation. SRF ChIP qPCR data from the *Srf* WT jejunum showed that SRF binding activity was significantly increased on the second CArG boxes including a well-conserved SRF binding motif, CCTTAAAAGG, in the first intron (I1) of *Dmpk* (Fig 2D). It also slightly increased SRF binding in the conserved CArG boxes in the promoter region (P1 and P2). The binding activities were mostly eliminated in the jejunum of *Srf* KO mice, suggestive of a SRF specific binding effect. Our transcriptome analysis [6] also confirmed that both jejunal and colonic SMC of WT mice expressed higher levels of *Dmpk* mRNA compared to whole intestinal tissue (Fig 2E). Furthermore, the expression of DMPK protein was significantly decreased in *Srf* KO jejunal muscle confirming its regulation by SRF (Fig 2F).

### SRF-induced DMPK may regulate RNA-binding proteins CELF1 and MBL1

**Fig 3:**
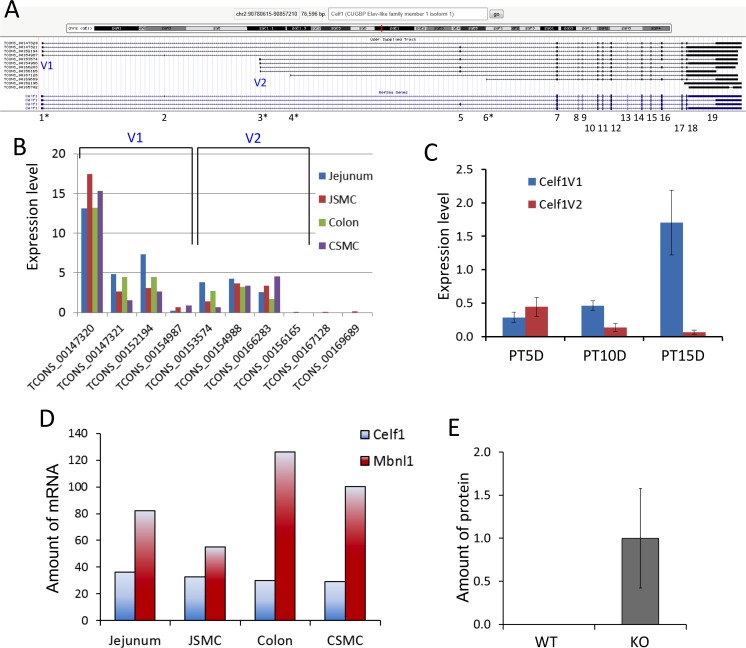
Altered expression of *Celf1* transcriptional variants in *Srf* KO SMC. (A) A genomic map of *Celf1* variants expressed in jejunum SMC. Exons are numbered 1–19. Four exons containing alternative transcription start sites are shown by a star (*). Two variable regions (V1 and V2) with the predominant transcriptional start sites are indicated. (B) Expression levels of *Celf1* variants in SMC and jejunal and colonic tissue, obtained from RNA-seq data [6]. (C) qPCR data showing expression changes of *Celf1* V1 and V2 transcripts at KO days 5, 10, and 15 (n = 3). (D) Comparison of expression levels of *Celf1* and *Mbnl1* in WT jejunum, colon, and their isolated SMC, obtained from RNA-seq data [6]. (E) Induction of CELF1 protein in KO muscle. Amount of protein expression was obtained from LS-MS/MS proteomics data.

Since SRF regulates DMPK in SMC, we hypothesized that alteration of L-type calcium channel expression by DMPK, resembling the regulatory pathway of the myotonic dystrophy model [14], is responsible for the functional changes of *Srf* KO SMC. DMPK functions to protect against muscular dystrophies by balancing the antagonistic activities of two RNA-binding proteins, CUGBP1 (CELF1) and MBNL1, whose dysregulation can lead to abnormal splicing of ion channels, including the voltage-gated calcium channel [14]. In muscular dystrophies, mutated *Dmpk* mRNAs containing long CUG repeats induce CELF1 expression, leading to the suppression of MBNL1 [28]. Therefore, we analyzed our transcriptome and proteomics data to see if there was a similar dynamic between these key proteins in SMC of *Srf* KO mice. Analysis of *Celf1* gene expression in jejunal SMC revealed the presence of 10 alternative transcriptional variants that encoded up to 19 exons [6] (Fig 3A). These variants consisted of two major groups: V1 representing long and short variants starting at exon 1 and V2 representing a cryptic exon 3. Group V1 was expressed more than V2 in both jejunal and colonic SMC, and the predominant transcriptional variant was TCON_00147320 of group V1 (Fig 3B). Notably, *Celf1* expression was significantly induced at PT15D in *Srf* KO mice although V1 and V2 were differentially expressed: V1 began to increase as early as PT10D, whereas V2 expression began to decrease at PT10D (Fig 3C). Both V1 and V2 variants appear to encode open reading frames with 420–514 amino acids. Consistent with the muscular dystrophy model, *Mbnl1* mRNAs levels were typically elevated 2–3 times higher than *Celf1* in jejunal and colonic SMC (Fig 3D). The proteomic analysis also showed that CELF1 expression was significantly induced in *Srf* KO but undetectable in WT mice (Fig 3E).

### Functional changes in intracellular Ca^2+^ transients and contractions in *Srf* KO SMC

**Fig 4:**
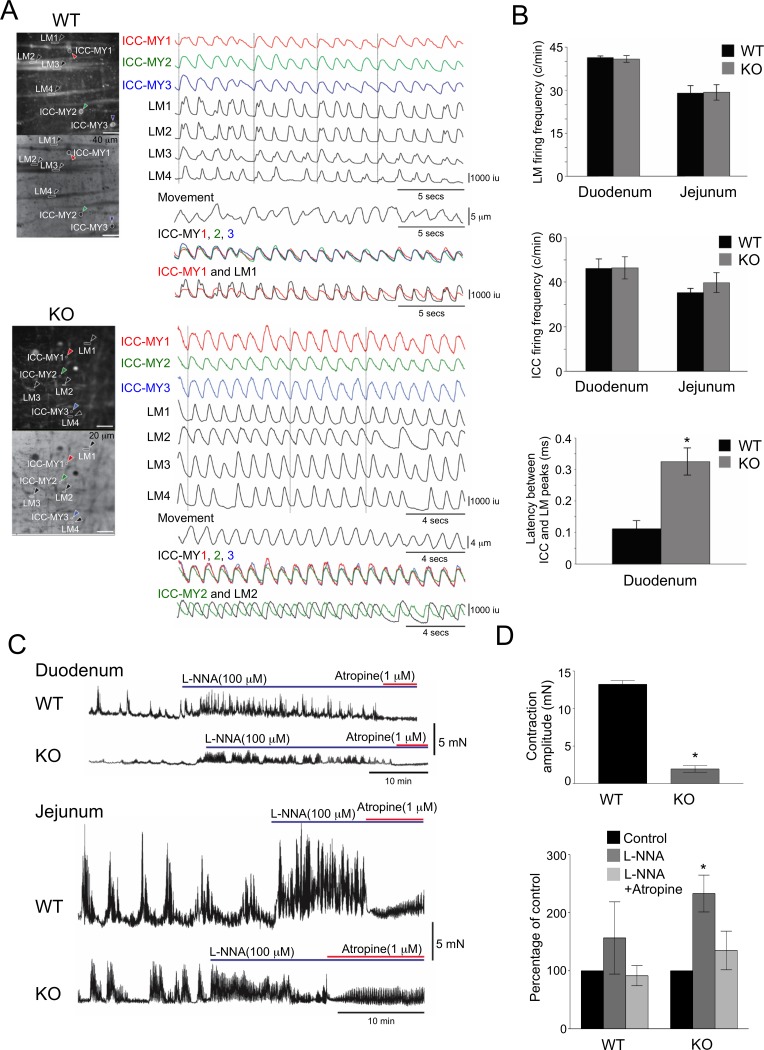
Uncoupling of Ca^2+^ transients between SMC and ICC resulting in reduced intestinal contractility in *Srf* KO mice. (A) Ca^2+^ transients recorded from several longitudinal SMC (LM) and ICC-MY in WT and KO jejunum within a field of view. Note that Ca^2+^ transients in ICC-MY (overlay of ICC-MY1-3) occur almost synchronously in WT and KO. Ca^2+^ transients in ICC-MY and LM are in phase in WT muscles (overlay of ICC-MY1 and LM1). Reduced coupling and greater latency was observed between the Ca^2+^ transients in ICC-MY and SMC in KO muscles (overlay of ICC-MY2 and LM2). (B) Frequency of Ca^2+^ transients in intestinal LM, frequency of Ca^2+^ transients in ICC-MY, and latency between Ca^2+^ transients in ICC-MY and LM of WT and KO mice are shown in graphs. (C) Contractions of WT and KO duodenal and jejunal muscles measured before and after L-NNA (100 μm) and atropine (1 μm). (D) Comparison of jejunal contractile amplitudes and summary of L-NNA and L-NNA plus atropine effects on jejunal contractions in WT and KO (expressed as percentages of control activity, i.e., no drugs present; areas under the curve for WT or KO in C). * *p* ≤ 0.5, n = 5

To investigate the effects of *Srf* KO in SMC on intestinal contraction, we measured the transient intracellular levels of Ca^2+^ ([Ca^2+^]_i_) in pacemaker (ICC-MY) and longitudinal muscle (LM) cells (S1 and S2 Videos). In the WT duodenum and jejunum, we observed spontaneous Ca^2+^ transients in ICC-MY that consistently spread to SMC in LM (S1 Video and Fig 4A). In muscle cells, Ca^2+^ transients were synchronized within muscle fiber clusters indicating their causal relationship with muscle action potentials [29]. Although Ca^2+^ transients in ICC-MY appeared normal in *Srf* KO duodenal and jejunal preparations (S2 Video and Fig 4B), longitudinal muscle cells in 60% of *Srf* KO duodenal and jejunal segments exhibited an extended latency in Ca^2+^ transients signifying a delay in SMC activation (Fig 4A and 4B). These observations suggested that in *Srf* KO GI muscles, the propagation of pacemaker activity from ICC-MY and/or L-type calcium channel activated action potentials were disrupted [24, 29].

Compared to WT, *Srf* KO duodenal and jejunal muscles also displayed smaller amplitudes during phasic contractions, which represented the mechanical activity of muscle (Fig 4C and 4D). Blocking of nitric oxide synthesis with L-NNA (100 μm) increased the amplitude of contractions in WT and *Srf* KO mice, and blocking of cholinergic input to the muscle with atropine (1 μm) reduced phasic contractions demonstrating that spontaneous inhibitory and excitatory neural inputs were intact in KO muscles (Fig 4C and 4D).

### Identification of multiple transcriptional variants of the calcium channels expressed in SMC

**Fig 5:**
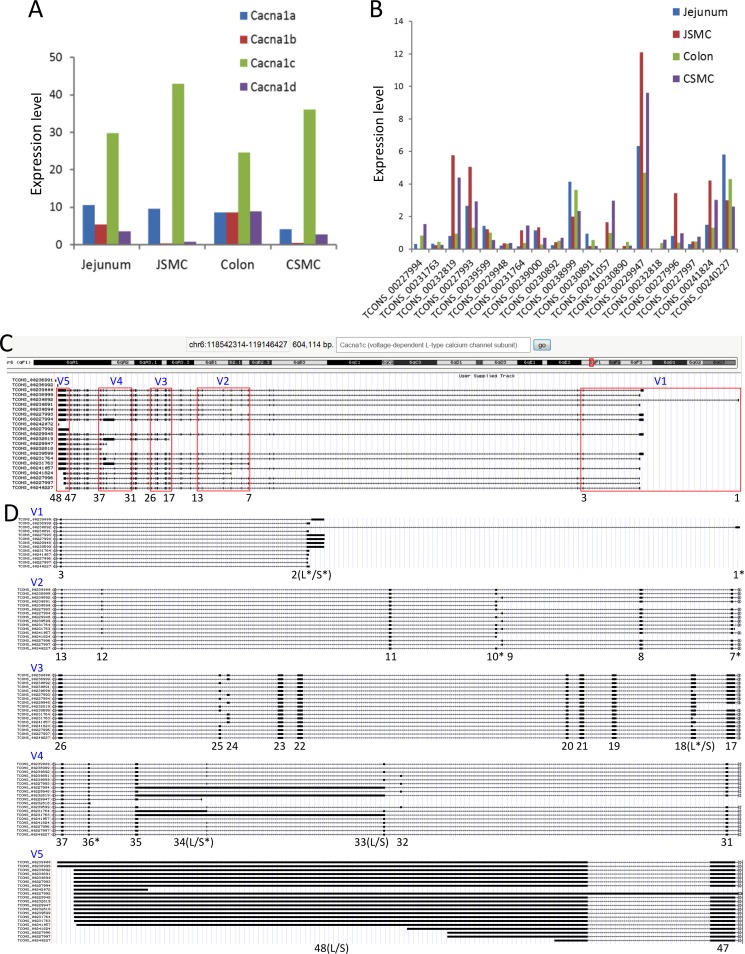
Identification of a predominant subtype and alternative transcriptional variants of L-type calcium channels expressed in SMC. (A) Expression levels of L-type calcium channel subtypes in SMC of jejunum and colon. (B) Expression levels of *Cacna1c* variants in SMC and tissue of jejunum and colon. (C) A genomic map of *Cacna1c* variants. Five variable regions (V1-5) are indicated. Exons are numbered 1–48. (D) Magnified view of variable regions showing alternatively started or spliced exons (indicated as exon numbers). Seven exons containing alternative transcriptional start sequence are shown by a star (*). Long (L) and short (S) exons that are differentially started or spliced are indicated.

**Fig 6:**
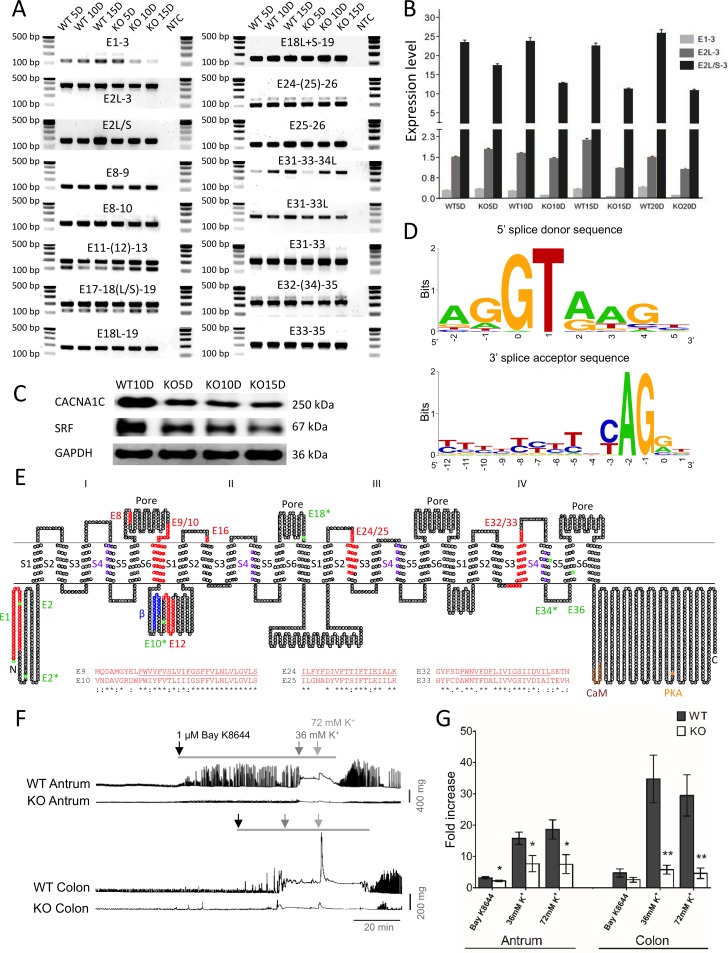
Alternations of L-type calcium channel transcripts, protein, and action potential in KO smooth muscle. (A) PCR validation of alternatively started and spliced exons of *Cacna1c* in WT and KO jejunum smooth muscle at KO days 5, 10, and 15. NTC is non template control. Primer sets were designed from variant exons in the regions (e.g. E1-3, forward primer spanning a region on exon 1 and reverse primer on exon 3). (B) qPCR data showing decreased expression of *Cacna1c* variants starting at exon 1 and exon 2 long and short forms (E1-3, E2L-3, and E2L/S-3) at KO days 5, 10, 15, and 20. E1-3, a region spanning exons 1 and 3; E2L-3, a region spanning exon 2 long (L) form and exon 3; E2L/S-3, a region spanning exon 2 long (L) and short (S) forms and exon 3. (C) Western blot showing decreased levels of CACNA1C protein in *Srf* KO muscle. (D) Consensus sequence of 5’ splice donor and 3’ splice acceptor sites. (E) A topological map of CACNA1C variants. Amino acid sequence is written in small circles. Four motifs are indicated as I-IV and six transmembrane domains, S1-S6. Four pore regions are also indicated. Colors on amino acid sequence show particular regions and domains: red, missing or inserted peptides from differentially spliced exons; purple, voltage sensors in S4 transmembrane domains; green, start codons found in differentially spliced variants (*, start codons deduced from indicated exons that are differentially spliced; blue, β subunit binding domain; brown, CaM (calmodulin) binding domain; orange, PKA (protein kinase A) phosphorylation site. Alignment of alternatively spliced exons E9/10, E24/25, and E32/33 are shown. (F) Isometric force recordings from antrum and colon of WT and KO mice. Bay K8644 (1 μM) and high potassium (K^+^) Krebs (36 mM and 72 mM) were applied to the tissues (indicated by bar and arrows). (G) The graph summarizes the results for 9 antral and 5 colonic WT and KO tissues. The responses to Bay K8644, 36 mM K^+^, and 72 mM K^+^ were significantly decreased in KO antrums, and the responses to 36 mM K^+^ and 72 mM K^+^ were significantly reduced in KO colons compared to WT. * and ** represent *p* ≤ 0.05 and *p* ≤ 0.01 respectively.

To investigate whether decreased contractile activity may be due to a reduction of L-type calcium channel activated action potentials in *Srf* KO SMC, we examined closely the expression profile of L-type calcium channels in SMC. We previously acquired the transcriptomes of primary SMC isolated from the jejunum and colon, which contained genome-scale expression data including the absolute numbers of gene isoforms [6]. Our analysis of the jejunal and colonic SMC transcriptomes revealed expression of four L-type calcium channel subunits: *Cacna1a*, *Cacna1b*, *Cacna1c*, and *Cacna1d* (Fig 5A). *Cacna1c*, the predominantly expressed subunit (Fig 5A), was differentially expressed as multiple transcriptional variants in jejunal and colonic SMC (Fig 5B). Fig 5C shows the genomic map of the 22 *Cacna1c* variants as displayed on the UCSC Smooth Muscle Genome Browser that we have previously built [6] (Fig 5C). There were five regions of the *Cacna1c* gene (V1-5) that contained alternative start sites at exons 1, 2, 7, 10, 18, 34, and 36 (Fig 5D). The alternatively initiated and differentially spliced exons were validated by PCR using variable exon-specific primer sets for *Srf* KO jejunum and WT cDNAs at post-tamoxifen (PT) injection days, 5, 10, and 15 (Fig 6A). All PCR amplicons matched the length of expected exon variants. qPCR analysis showed that the 7 *Cacna1c* variants starting at the short exon 2 (TCONS_00238999, TCONS_00230891, TCONS_00231764, TCONS_00241057, TCONS_00227996, TCONS_00227997, and TCONS_00240227) [6] were predominantly expressed in smooth muscle (Fig 6B).

### Reduced contractility is linked to down-regulation of L-type calcium channels

Interestingly, transcripts starting on exon 1 and exon 2 (with long and short variations) were significantly decreased in *Srf* KO smooth muscle. More specifically, transcriptional variants starting from the short exon 2 began to decrease in smooth muscle at post-tamoxifen day 5 (PT5D) while those with exon 1 and long exon 2 began to decrease at PT10D (Fig 6B). The reduction in mRNA expression led to the decrease in protein levels, and as hypothesized, CACNA1C protein levels decreased significantly and gradually during PT5D-15D as SRF was further abolished (Fig 6C). Examination of the 5’ splice donor and 3’ splice acceptor sequences of the 48 exons in the 22 variants also revealed that the donor and acceptor sites were well conserved with 46 canonical dinucleotides GT and AG in addition to 2 non-canonical GC-AG splice site pairs (Fig 6D). Furthermore, the full-length *Cacna1c* transcripts consisted of 13 variants, which differentially started at exon 1 (1 variant) and exon 2 (5 long variants and 7 short variants, Fig 5C and 5D).

Amino acid (aa) sequences of all 22 *Cacna1c* transcriptional variants were further analyzed by aligning all putative variant protein sequences. Most variants encoded for proteins ranging from 771 to 2169 aa in length. The longest variant was TCONS_00230892 (starting from exon 1), which was 2169 aa in length with an additional 46 aa at the N-terminus. This variant consisted of 4 homologous motifs (I-IV), which each contained 6 transmembrane domains, including an S4 voltage sensor and a pore region. Interestingly, 9 of the variants were full-length proteins with the same 4 motifs. Moreover, the full-length variants also contained channel regulatory domains: a β subunit binding domain (βBD) on a cytoplasmic loop between motif I and II, a calmodulin binding domain (CaMBD), and a protein kinase A phosphorylation site (PKAp) at the C terminus. The channel topology of CACNA1C protein showed insertion or deletion of amino acid (aa) sequences in several exons (Fig 6E). Alternatively started or spliced exons resulted in truncated proteins (exons 2, 10, 18, 34, and 36) as well as deletion or insertion of aa sequences in different domains (exons 8, 9, 10, 12, 16, 24, 25, 32, and 33). Interestingly, three sets of two alternatively spliced exons E9/10, E24/25, and E32/33 redundantly encoded two similar hydrophobic transmembrane domains of IS6, IIIS2, and IVS3, respectively (Fig 6E). Among the seven predominately expressed cDNA variants of Cacna1c differentially starting on short exon 2, five variants (TCONS_00238999, TCONS_00230891, TCONS_00241057, TCONS_00227996, and TCONS_00227997) [6] encoded a full-length protein containing all four motifs and regulatory domains. The loss of these full-length variants was most likely responsible for the significant reduction of CACNA1C protein in the *Srf* KO smooth muscle (Fig 6C).

To measure the changes in L-type calcium channel-mediated contractile activity in WT and *Srf* KO GI smooth muscles, we recorded the isometric forces of the WT antrum and colon during spontaneous contractions. Bath application of Bay K8644, an L-type calcium channel agonist, caused a marked increase in the contractile activity in both colon and antrum WT muscle strips. Contractile activity was determined by measuring the area under the trace (AUT, Fig 6F). In contrast, the Bay K8644-induced increase in contractile activity was significantly less in KO antrum (Fig 6F). In colon, the increase in contractile activity was also less in KO animals compared to WT, although this was not statistically significant (Fig 6F).

We also utilized a high potassium (K^+^, 36 mM and 72 mM) Krebs solution to depolarize the muscle tissues, which subsequently opens voltage-dependent L-type calcium channels and initiates contraction. High K^+^ Krebs caused a large sustained contraction in WT antrum and colon (Fig 6F). In contrast, the contractile response of the KO antrum and colon to high K^+^ Krebs was significantly decreased (Fig 6F). The bar graph in Fig 6G summarizes the fold changes in AUT in response to Bay K8644 and high K^+^ Krebs in WT and KO antrum and colon. It clearly illustrates the significantly decreased contractile responses in *Srf* KO antrum and colon tissues. We also performed the same experiments with jejunal tissue. The reduced responses in the KO jejunum, however, were variable and not statistically significant (data not shown). We decided to calculate fold increase, rather than using absolute contraction magnitude, because this will take into account that KO animals have a reduced smooth muscle mass to begin with.

Collectively, our data suggests the SRF induced expression of *Dmpk* via the conserved CArG boxes specifically in SMC. In the absence of SRF in *Srf* KO mice, SMC decreased expression of the *Dmpk* gene, which abnormally switched the preferential expression of MBNL1 to CELF1. This preferential expression of CELF1 over MBNL1, which mirrors that of the myotonic dystrophy model [14], most likely triggers the abnormal splicing of L-type calcium channels resulting in the reduced contractility in the *Srf* KO smooth muscle.

## Discussion

In this transgenic animal study, we found that the transcription factor SRF may regulate DMPK, a muscle-specific protein kinase that may control expression of an L-type calcium channel smooth muscle isoform. Not surprisingly, the ablation of SRF in SMC led to greatly decreased contractile activity and significantly delayed intracellular Ca^2+^ transients. Interestingly, the reduced contractility was linked to down-regulation of L-type calcium channels, which corresponded to reduction of DMPK, a newly discovered potential SRF target. In this study we demonstrated the critical role of SRF in the control and maintenance of intracellular Ca^2+^ transients as well as contractility in GI SMC.

In the proteomics data, we identified 82 proteins whose expression was affected by SRF. The IPA software recognized 16 proteins (9 direct and 7 indirect relationships with SRF) that had been previously reported (Fig 2A). We also examined SRF-binding sites (SRF ChIP data available in UCSC ENCODE) in the genes of the SRF-induced proteins identified in this study (S3 Table), and we built a CArGome search browser using UCSC Genome Browser that enabled us to identify CArG boxes within SRF binding sites. The CArGome search browser was updated with transcriptome data of primary SMC isolated from colon and jejunum (UCSC Smooth Muscle Genome Browser) [6]. The browser identified multiple splice variants of *Dmpk* expressed in SMC, an SRF binding site, and a CArG box (Fig 2B), which confirmed our proteomics data (Fig 2F).

DMPK is of particular interest because its expression is restricted to muscles, including smooth muscle, and is directly linked to muscular diseases such as myotonic dystrophy [16]. Interestingly, our findings suggest that the binding of SRF to CArG box at intron 1 (Fig 2B) may regulate the specificity of *Dmpk* expression in SMC (Fig 2D). However, to ascertain the SRF/CArG box-mediated regulation of *Dmpk* expression in SMC, further anlaysis using techniques such as CRISPR/Cas9 genome editing of the CArG box *in vivo* is required. In this context, genome editing of a single CArG box has been shown to dramatically reduce *Cnn1* expression in mice, providing definitive *in vivo* evidence for a direct role of SRF in controlling target gene expression [30]. Nevertheless, our findings are consistent with a recent study, which suggested that DMPK is implicated in myotonic dystrophy by its dysregulation of MBNL1 and CELF1 mediated alternative splicing of the L-type calcium channel CACNA1S [14]. The latter then leads to altered gating properties adversely affecting contractility of muscles.

Remarkably, CACNA1C protein was reduced in *Srf* deficient smooth muscle as early as 5 days post tamoxifen injection (Fig 6C). However, the UCSC Smooth Muscle Genome Browser showed that there were no SRF binding sites associated with the *Cancna1c* gene. Since SRF binding sites from ENCODE data in the UCSC genome browser were obtained from SRF ChIP-seq data of C2C12 skeletal myocytes, this discrepancy may be explained by the silencing of the *Cacna1c* gene in the C2C12 myocytes since *Cacna1c* actually encodes 19 CArG boxes that are conserved between mice and humans. Moreover, although SRF uses the same CArG boxes to program SRF-dependent genes in skeletal, cardiac, and smooth muscle cells [31], there are some genes that are exclusively expressed in each muscle cell type. For example, skeletal muscle cells express L-type calcium channel subtype CACNA1S but not CACNA1C [32]. Thus, further experimental validation is needed to demonstrate whether any of the CArG boxes associated with the *Cacna1c* gene may be functional and able to regulate gene expression.

*Cacna1c*, previously known as the cardiac isoform [33], is actually the dominant isoform of the L-type calcium channel in SMC (Fig 5A). The gene is expressed as 22 multiple transcriptional variants in SMC (Fig 5B and 5C). Interestingly, the *Cacna1c* transcriptional variants and CACNA1C protein levels significantly diminished in *Srf* KO smooth muscle (Fig 6B and 6C) as DMPK expression decreased and CELF1 expression increased (Fig 2F and Fig 3E). Consistent with these findings, the L-type calcium channel action potentials induced by both the agonist and membrane depolarization showed that this channel activity was largely abolished in KO SMC (Fig 6F). Therefore, the disruption of L-type Ca^2+^ channel expression may result in decreased amplitude of action potentials, which may uncouple the Ca^2+^ transients between SMC and ICC resulting in decreased intestinal contractility in *Srf* KO mice (Fig 4D).

**Fig 7:**
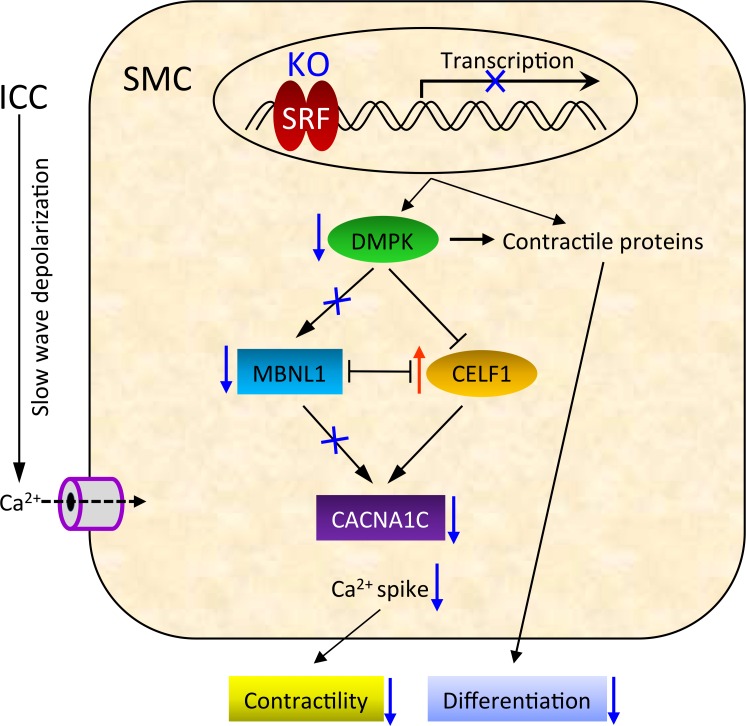
Model showing the possible molecular pathways by which SRF regulates contractility via DMPK and CACNA1C in SMC.

From our data, we propose that SRF has a regulatory role in both the contractility and differentiation of SMC (Fig 7). Accordingly, loss of SRF expression in SMC results in decreased expression of SRF-dependent proteins, including contractile proteins and DMPK. During early cellular changes in pathophysiologic conditions, the reduction of contractile proteins may interfere with SMC differentiation. Furthermore, the resultant decrease in DMPK expression may switch the preferential expression of MBNL1 to CELF1, which may suppress expression of the L-type calcium channel CACNA1C. The reduced expression of CACNA1C in SMC may then decrease the coordination between pacemaker ICC and SMC to negatively impact intestinal motility. Our data, therefore, provides new insights into how hypo-contractile conditions may pathologically develop within the GI tract and augments the overview of conventional SRF regulation.

## Supporting information

S1 Table Oligonucleotides used in this study. (DOCX)

S2 Table A list of proteins that were down-regulated in the jejunal smooth muscle of *Srf* KO mice. (XLSX)

S3 Table Analysis of SRF binding sites and CArG boxes of SRF down-regulated protein genes. (XLS)

S1 Video Ca^2+^ transients and tissue movement activities recorded from longitudinal smooth muscle cells and ICC-MY in *Srf* WT jejunum within a field of view. *QuickTime Player is required.
(MOV)

S2 Video Ca^2+^ transients and tissue movement activities recorded from longitudinal smooth muscle cells and ICC-MY in *Srf* KO jejunum within a field of view. *QuickTime Player is required.
(MOV)

## Abbreviations

SRF: serum response factor
SMC: smooth muscle cells
CArG box: [CC (A/T)_6_ GG
CArGome: genome-wide CArG boxes
ICC: interstitial cells of Cajal
MY: myenteric
LM: longitudinal muscle
GI: gastrointestinal
[Ca^2+^]_I_: intracellular calcium concentrations
CACNA1C: α1C subunit of L-type Ca^2+^ channel
DMPK: myotonic dystrophy protein kinase
CACNA1S: α1S subunit of L-type Ca^2+^ channel
KO: knockout
WT: wild type
RT-PCR: reverse-transcription polymerase chain reaction
qPCR: quantitative PCR
PT: post-tamoxifen
aa: amino acid
βBD: β subunit binding domain
CaMBD: calmodulin binding domain
PKAp: protein kinase A phosphorylation
