# Supplementary material for: Serum response factor regulates smooth muscle contractility via myotonic dystrophy protein kinases and L-type calcium channels
Source: PLoS One. 2017 Feb 2;12(2):e0171262. doi: 10.1371/journal.pone.0171262 (PMC5289827; doi:10.1371/journal.pone.0171262)
Supplement: S1 Table — (DOCX) [file pone.0171262.s001.docx]

**S1 Table.** **Oligonucleotides used in this study.**

1. Genotyping

| **Set** | **Name** | **Sequence (5' to 3')** | **Size (bp)** | **Region** |
| --- | --- | --- | --- | --- |
| 1 | SRF-gt1 | GCTGCTTACTGGAAAGCTCATGG | 128(WT)/428(KI) | lox insertion |
|  | SRF-gt1r | GCTGGTTTGGCATCAACTTGACAC |  |  |
| 2 | Myh-I1 | GTTCTGACCCCATCTCTTCACTCC | 232(WT)/~280(Cre) | Myh11 I1 |
|  | Myh-E2r | ACCAACTCCACGACCACCTCATC |  | Myh11 E2 |
|  | Cre-r | GTAGTCCCTCACATCCTCAGGTTC |  | CreERT2 insertion |
| 3 | SRF-gt1 | GCTGCTTACTGGAAAGCTCATGG | ~3 kb(WT)/390(KO) | lox deletion |
|  | SRF-gt2r | CTCCTCATCTCCAAACCTTCTGCTC |  |  |

1. RT-PCR/qPCR reactions

| Gene | **Forward (5' to 3')** | **Reverse (5' to 3')** | **Exons** | **Size(bp)** | **Region** |
| --- | --- | --- | --- | --- | --- |
| Cacna1c | CAAGGGCAAGGTGGTACATGAAGC | CAATGCAGCTGCAGGACTTGCTC | E1-3 | 125 | V1a |
| Cacna1c | GTACTGAGGATGCATTACAATCTCAC | CAATGCAGCTGCAGGACTTGCTC | E2L-3 | 364 | V1c |
| Cacna1c | GCTGCATTTCTTCCTCTTTGTGGC | CAATGCAGCTGCAGGACTTGCTC | E2L/S | 169 | V1d |
| Cacna1c | GCTGACGGTGTTCCAGTGTATCAC | GTCAGTCTGGTCATCTTTGGATCC | E8-9 | 121 | V2a |
| Cacna1c | GCTGACGGTGTTCCAGTGTATCAC | CTTGGTTCTCGGTGTTCTTAGCGG | E8-10 | 169 | V2b |
| Cacna1c | CAAAGGCTACCTGGACTGGATCAC | GAGCATGCCCACAAGTGAGACTG | E11-(12)-13 | 188 | V2c |
| Cacna1c | CAGTCTCTCCTCACTGTGTTTCAG | CATTGCGGTGGACAACCTGGCTGA | E17-18(L/S)-19 | 204 | V3-1 |
| Cacna1c | TGTCATTCAGATCCTGACCGGGGA | CATTGCGGTGGACAACCTGGCTGA | E18L-19 | 180 | V3-3 |
| Cacna1c | CTGGAATTCGGTGATGTATGATGGG | CATTGCGGTGGACAACCTGGCTGA | E18L+S-19 | 153 | V3-4 |
| Cacna1c | CCACCATTTTCACCATTGAAATTGCTC | CTTCAATATCCTGGACCTGCTGG | E24-(25)-26 | 165 | V3c |
| Cacna1c | CCTAGGCAATGCAGACTATGTCTTC | CTTCAATATCCTGGACCTGCTGG | E25-26 | 131 | V3d |
| Cacna1c | CTATGGCCAGAGCTGCCTCTTC | CAACCCTCCAGACCACATTTCCAG | E31-33-34L | 257 | V4f |
| Cacna1c | CTATGGCCAGAGCTGCCTCTTC | GCCTGCTTAACATCCATCTGTTTCC | E31-33L | 168 | V4c |
| Cacna1c | CTATGGCCAGAGCTGCCTCTTC | CTGAACATACCCAATGCTCTCCCTC | E31-33 | 222 | V4d |
| Cacna1c | CTATGGCCAGAGCTGCCTCTTC | GGGTTACTTTAGTGATCCCTGGAATG | E32-(34)-35 | 134 | V4a |
| Cacna1c | CTATGGCCAGAGCTGCCTCTTC | GACGCCTTGATTGTTGTGGGTAGC | E33-35 | 163 | V4b |
| CelfV1 | AGCAAGGCAGCAGCTGAGGCAG | GCATCAAGATCTGGTTGGTCTGGG | E1-5 | 305/252/100 |  |
| CelfV2 | GCATCAAGATCTGGTTGGTCTGGG | CATCAAGATCTGGTTGGTCTGGGTG | E3-5 | 276/124 |  |
| Uba1 | CTTGACTTCGGCTCCTTGAGGAG | CAGTTAGAACCCGGCTTTGGATCAG | E1-2 | 124 |  |
| Srf | ATCTGACAGCAGTGGGGAAA | TCTGGATTGTGGAGGTGGTAC | E2-3 | 86 |  |
| Acta2 | CAGAGACTCTCTTCCAGCCATCTTTC | CGTTGTTAGCATAGAGATCCTTCCTG | E7-8 | 119 |  |
| Ubb | ATTCGGTCTGCATTCCCAGT | AATTGGGGCAAGTGGCTAGA | E2 | 65 |  |

1. ChIP qPCR analysis

| **Name** | **Forward (5' to 3')** | **Reverse (5' to 3')** | **CArG sequence** | **Size(bp)** | **Region** |
| --- | --- | --- | --- | --- | --- |
| Dmpk1 | GCTAACTTGTCGCCTGGCTTGC | CCACATTCCTGCCTGGGCTAG | CCTAAAATGT | 92 | Promoter(P) 1 |
| Dmpk2 | CTAGCCCAGGCAGGAATGTGG | GAGGCAAGTGGTGGATCCAGG | GCTATTTTGG | 98 | Promoter(P) 2 |
| Dmpk3 | ACCCTCTGGGAAGGAGCAGTC | GGAGAGAGCCAAGGCGTTGAG | CCTTTTGTGG | 131 | Intron(I) 1 |
|  |  |  | CCTTAAAAGG |  |  |
| Dmpk4 | CAGCATCCCATGCTCCTTAGAGC | CTCAGCACCTTGGCTGCCTTGC |  | 92 | Intron(I) 9 |
| Dmpk5 | GGTGGAGAAAAGAGGAGGAGGC | CCAGAACCCTAGCTGCATGTCTG |  | 94 | Exon(E) 1 |
